# Supplementary material for: txtools: an R package facilitating analysis of RNA modifications, structures, and interactions
Source: Nucleic Acids Res. 2024 Mar 21;52(8):e42. doi: 10.1093/nar/gkae203 (PMC11077046; doi:10.1093/nar/gkae203)
Supplement: gkae203_Supplemental_File [file gkae203_supplemental_file.pdf]

Supplementary Figures

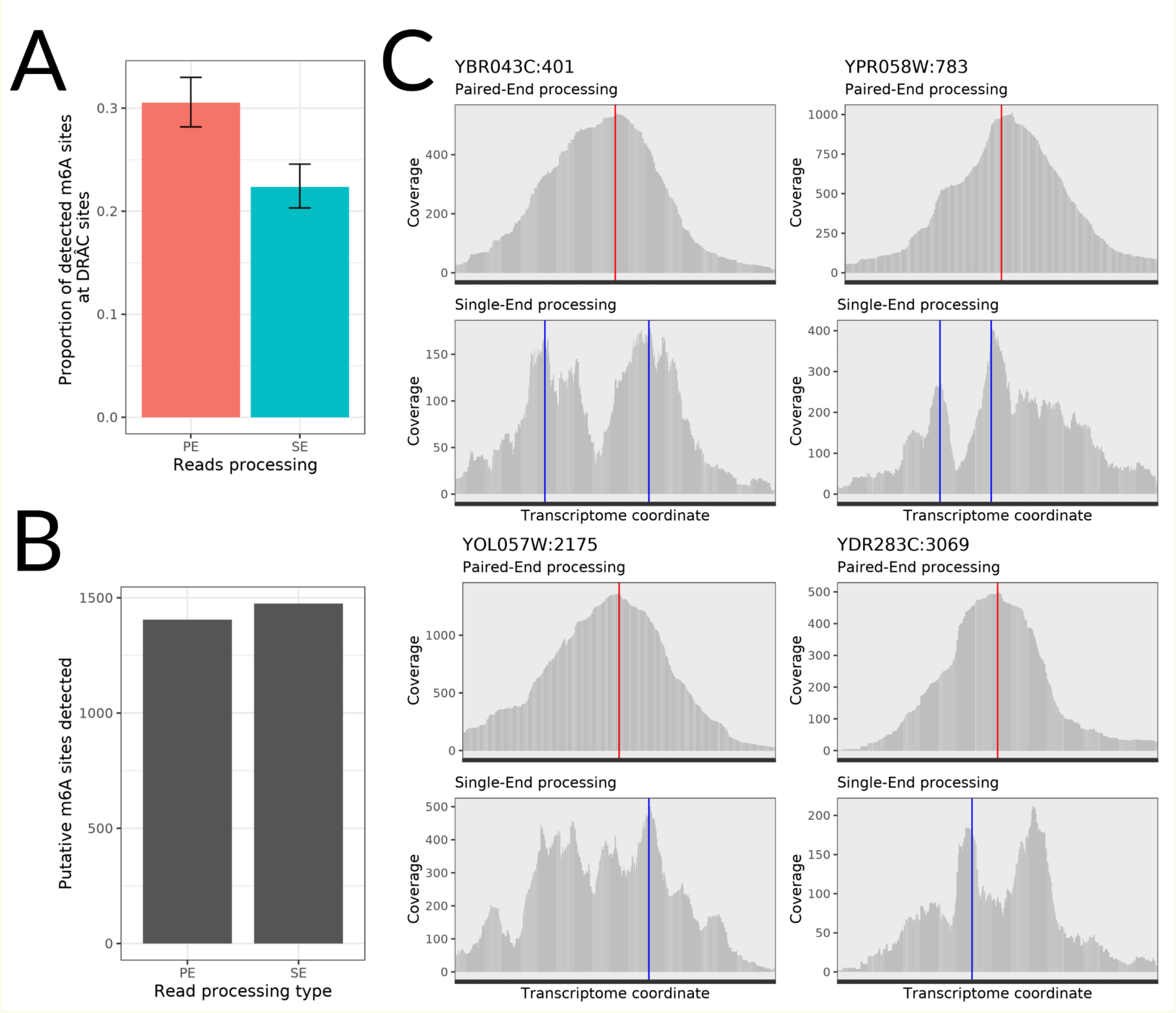

Supplementary Figure S1. A) Barplot showing the proportion of detected putative m6A sites that fall exactly on a DRAC motif using either the paired-end reads (PE) or the single-end reads (SE) processing options of txtools. Error bars show the binomial confidence interval at 95% confidence. B) Barplot showing the number of detected putative m6A sites using either the paired-end reads (PE) or the single-ends (SE) reads processing options of txtools. C) txtools' coverage plots showing the aggregated coverage of the WT_IP replicates for four selected putative m6A sites detected using the paired-ends processing strategy and marking the putative m6A site with a red line (on top), compared to the same site's coverage using the single-end processing strategy with detected putative m6A sites marked with blue lines (bottom). Each site is defined by the gene and their relative transcriptomic coordinate as gene:txcoor.
